# Relation between awareness of circulatory disorders and smoking in a general population health examination

**DOI:** 10.1186/1471-2458-6-48

**Published:** 2006-02-27

**Authors:** Ulrich John, Christian Meyer, Monika Hanke, Henry Völzke, Anja Schumann

**Affiliations:** 1 University of Greifswald, Institute of Epidemiology and Social Medicine, Walther-Rathenau-Str. 48, D-17487, Germany

## Abstract

**Background:**

Little is known about proportions of smokers who maintain smoking after they are aware of a circulatory disorder. The goal was to analyze the extent to which the number of circulatory disorders may be related to being a current smoker.

**Methods:**

Cross-sectional survey study with a probability sample of residents in Germany investigated in health examination centers. Questionnaire data of 3,778 ever smoking participants aged 18 – 79 were used, questions included whether the respondent had ever had hypertension, myocardial infarction, other coronary artery disease, heart failure, stroke, other cerebrovascular disease, peripheral vascular disease, and venous thrombosis. Logistic regression was calculated for circulatory disorders and their number with current smoking as the dependent variable, and odds ratios (OR) are presented adjusted for physician contact, inpatient treatment, smoking cessation counseling, heavy smoking, exercise, overweight and obesity, school education, sex and age.

**Results:**

Among ever smokers who had 1 circulatory disorder, 52.1 % were current smokers and among those who reported that they had 3 or more circulatory disorders 28.0 % were current smokers at the time of the interview. The adjusted odds of being a current smoker were lower for individuals who had ever smoked in life and had 2 or more central circulatory disorders, such as myocardial infarction, heart failure or stroke, than for ever smokers without central circulatory disorder (2 or more disorders: adjusted OR 0.6, 95 % confidence interval, CI, 0.4 to 0.8).

**Conclusion:**

Among those with central circulatory disorders, there is a substantial portion of individuals who smoke despite their disease. The data suggest that only a portion of smokers among the general population seems to be discouraged from smoking by circulatory disorders or its accompanying cognitive or emotional processes.

## Background

Circulatory disease prevention includes smoking cessation [1]. Although awareness of having circulatory disease prima facie should be assumed to function as a threat to the smoker sufficient in strength to stimulate smoking cessation [2] there is evidence that diseased smokers are even less likely to quit than non-diseased smokers [3].

Among individuals with circulatory disorders, there are considerable proportions of current smokers. In a general population survey carried out in 1997 in different areas of Finland [4], there appeared to be no differences in smoking status by awareness of hypertension. Among male ever smokers aged 25 to 64 with hypertension (> 160 mm Hg systolic or > 95 mm Hg diastolic blood pressure) who were aware of hypertension but untreated, there were 55.6 % current smokers and among male ever smokers who had been unaware of hypertension and untreated, there were 54.2 % current smokers. Among those male ever smokers who reported current antihypertensive medication, the proportion of current smokers was 29.3 %. Among women, the current smoker proportions were 58.1 % of those aware but untreated, 61.1 % of those unaware and untreated, and 57.1 % of those with hypertensive medication. In a national survey carried out among the general population aged 16 or older living in England 1994, lower odds for awareness of hypertension were found for current smokers compared to never smokers [5].

Awareness of a tobacco-attributable disease might support smoking cessation depending on the degree the smoker feels vulnerable by a disease compared to nonsmokers and the strength of *threat*, i. e. perceiving the disease to be life-threatening [2]. The *number *and *type *of circulatory disorders might be major determinants of the strength of threat. According to type, one might argue that in the lay view diseases of organs close to the body site of tobacco smoke intake are considered more affected from smoking than peripheral organs [2,6]. Altogether, little detail is known about how many individuals smoke although they are aware of a circulatory disorder and about associations between the number and type of circulatory disorders with smoking status from general population studies.

The goals of the present paper were, first, to describe how many individuals are current smokers among those who are aware of circulatory disorder, second, to analyze relations of the number of circulatory disorders with smoking status and to account for variables potentially relevant in smoking cessation: physician contact, smoking cessation counseling, exercise, BMI, school education, age and gender in a probability sample representative of the general population aged 18 – 79 of Germany, a country for which data show little intention to quit among the smoking population [7]. Third, we analyzed whether having central circulatory disorders, such as myocardial infarction or stroke, predicted lower odds of being a current smoker among ever smokers whereas this might not be the case for peripheral circulatory disorders, such as peripheral vascular disease. We hypothesized that among ever smokers those with central circulatory disorders have lower odds of being a current smoker than ever smokers without these circulatory disorders.

## Methods

### Sample

A national sample of the German civilian, noninstitutionalized population aged 18 – 79 was used [8]. A random sample (N = 13,222) stratified by the 16 German Federal States was drawn from the German residents' registration files in which every resident's address, age and gender is included by law. On contact of the target persons, 1,621 (12.3 %) had been deceased, moved, were unknown under the registered address or did not speak German. Of the remaining 11,601 individuals, 7,124 (61.4 %) participated in the study [9]. They had been invited to take part in a health examination that was conducted in an examination center, i. e. rooms rented for the purpose of the study in 130 sample points [9,10]. Data were collected between October 1997 and March 1999 [9]. The participants gave written consent after they had been informed about the purpose and the data security provisions of the study and that they could withdraw consent at any time [8]. The data collection had been carried out under the auspices of the German Federal Ministry for Health. According to that, there was no ethical concern. The study was carried out in compliance with the Declaration of Helsinki [11]. Among the study participants, 6,963 had complete information on smoking status. Among them, 3,778 individuals were ever smokers (54.3 %). These constituted our final sample.

### Assessments

All data except body weight and height were gathered by a questionnaire to be filled in by the participant in the examination center. The participant was assisted if unable to fill in the questionnaire. A list of disease was presented to the respondent with the question "Which of the following diseases did you ever had?" The following circulatory disorders were included: "hypertension", "myocardial infarction", "disturbed coronary circulation, angina pectoris", "heart failure", "stroke", "circulatory disorder of the brain (only if coincided with palsy, disorders of feeling or speech and not due to migraine)", "disturbed blood flow of the legs, peripheral vascular disease, intermittent claudication", and "varicosity (varices, ulcus cruris)" The response categories were "Ever had: yes, no, do not know". We merged "no" and "do not know" into one category since only those disease conditions were considered which the respondents were definitely aware of. We defined hypertension, myocardial infarction, other coronary artery disease, heart failure, stroke or other cerebrovascular disease as central circulatory disorders, and differentiated them from peripheral circulatory disorders (peripheral vascular disease, venous thrombosis).

Smoking was assessed as part of the questionnaire. The individuals were asked whether they had formerly smoked or whether they currently smoked ("Did you formerly smoke or do you smoke currently?"). Current smokers answered whether they smoked daily or occasionally, i. e. less than daily. The number of cigarettes per day (cpd) or per occasion, cigars, and pipes was assessed for former smokers and the number of cpd, cigars, and pipes for current smokers each. Former smokers were those who had stopped smoking and were abstinent at the time of the health examination. Current smokers were those who said that they smoked currently at the time of the health examination. Ever smokers were defined as current smokers or former smokers. Never smokers were respondents who answered "Yes" to "never smoked yet (or tried very rarely to smoke)". Heavy smokers were defined as those who smoked 20 or more cpd. Consultation of a physician during the last 12 months prior to the health examination was assessed by the question "When did you consult a doctor (not a dentist) the last time ?" The categories were merged into "the last 12 months" and "more than 12 months ago". Inpatient treatment was assessed by the question "How many nights did you stay in a hospital as an inpatient during the last 12 months?" (no night, number of nights). Smoking cessation counseling was assessed for those who had consulted a physician during the last 12 months prior to the health examination by the question "Have you ever been counseled for smoking when visiting a doctor?" (yes, no). Exercise was assessed based on the questions "How often do you exercise?" (never, two hours per week or less, more than two hours per week). Body height was measured by a fixed stadia rod with the person wearing no shoes. The results were registered accurately to 0.5 centimeter. Body weight was measured by a scale with the person wearing no shoes and light clothing, and the results were registered accurately to 0.1 kg. Body weight of 150 kg or more was confirmed as valid in the data entry during the measurement. BMI was calculated as body weight in kilograms divided by body height in m^2^. We used BMI 18.50 to < 25.00 kg/m^2 ^for normal weight, BMI 25.00 to < 30.00 kg/m^2 ^for overweight and, due to small cell frequencies, merged all individuals with BMI > 30.00 kg/m^2 ^into the obesity category [12]. We analyzed six age groups (18–29, 30–39, 40–49, 50–59, 60–69, 70–79). School education was assessed by number of years at school based on the three-level German school system (9 or less, 10 – 11, 12 – 13 years).

### Statistical analysis

We calculated likelihood ratio chi^2 ^tests and used the effect size measure Cohen's w [13] to analyze bivariate associations between the presence of number of circulatory disorders and the proportion of current smokers among the ever smokers. According to Cohen [13] we interpreted values .10 or higher as indicating an effect. We applied logistic regression analyses with current smoking as the dependent variable for the analysis of the likelihood of being a current smoker among the ever smokers dependent on circulatory disorders, controlling for physician contact, inpatient treatment, smoking cessation counseling, heavy smoking, exercise, overweight and obesity, school education, sex and age, and we give the odds ratio (OR) and the 95 % confidence interval (CI). Since there were only 6 individuals that formerly and 8 individuals that currently smoked cigars or pipes only, the regression analysis that includes cpd was limited to cigarette smokers. All data analyses were performed with SPSS 13.0.

## Results

According to the bivariate data analysis, among those who said that they had one circulatory disorder, 52.1 % were current smokers, i. e. these individuals still smoked after the circulatory disorder had occurred (Table 1). Among those who said that they had 3 or more circulatory disorders 28.0 % were current smokers. According to single circulatory disorders, independent of the number of disorders that the respondent was aware of, the proportion of current smokers among ever smokers ranged from 29.7 % for myocardial infarction to 44.4 % for hypertension.

Among the ever smoking individuals, 83.2 % had started smoking at age 20 or younger. Those who had a circulatory disorder at age 20 or younger were 1.4 % of all ever smokers with a circulatory disorder. Thus, circulatory disorders occurred after the onset of smoking in the majority of the cases.

The multivariate data analysis using logistic regression analysis revealed that those individuals who had 2 or more central circulatory disorders had lower odds of being a current smoker compared to subjects without central circulatory disorders (Table 2). A second logistic regression analysis revealed that those with 2 or more central circulatory disorders did not have lower odds of being a current smoker than those with 1 central circulatory disorder (OR 0.8; CI 0.6 to 1.2). All OR were adjusted for physician contact, inpatient treatment, smoking cessation counseling, heavy smoking, exercise, overweight and obesity, school education, sex and age. Individuals who had consulted a physician and individuals who had been treated as inpatient showed lower odds of being a current smoker than subjects who did not have consulted a physician and subjects who had not been treated as inpatient. The adjusted OR for being a current smoker among ever smokers aged 18 to 49 was 1.0 (CI 0.8 to 1.4) for 1 central circulatory disease and 0.6 (CI 0.3 to 1.4) for 2 or more central circulatory diseases. Among those aged 50 to 79 the OR was 0.7 (CI 0.5 to 1.0) for one central circulatory disease and 0.6 (CI 0.4 to 0.9) for two or more central circulatory diseases (data not shown). A third logistic regression analysis in which the 8 single circulatory disorders were entered instead of the number of circulatory disorders in addition to the variables in the first logistic regression, revealed a significant OR only for "disturbed blood flow of the heart, angina pectoris". Individuals who ever had coronary artery disease showed an OR 0.7 (CI 0.5 to 0.96) to be a current smoker compared to those who declared themselves as normotensive. All OR were adjusted as in the first two logistic regression analyses (data not shown).

## Discussion

The study provides two main findings: First, for each of the single circulatory disorders, there were 29.7 % or more current smokers among those with a disease. With respect to the number of circulatory disorders, even among those who reported 3 or more, 28.0 % were current smokers. Second, lower odds to be a current smoker were found for individuals with central, but not with peripheral circulatory disease. The hypothesis was confirmed except that those with one central circulatory disorder did not show a relation with current smoking. Altogether, our data confirm former research that had found high proportions of current smokers among hypertensive individuals [4]. Our results were controlled for overweight and obesity and for exercise which were related to current smoking, supporting former findings [14,15].

Three approaches may explain the result of substantial proportions of smokers even among those who have suffered from diseases attributable to smoking: First, cognitive processes, such as denial, may be used by smokers to minimize or rationalize the health threat, i. e. finding a justification for the maintenance of smoking. The threat of disease might be related to further other psychological factors such as expectations to succeed in quitting smoking [2]. The reason is plausible also in the light of the finding that central, but not peripheral circulatory disease was related to the odds of being a current smoker. This finding supports former research [2,6]. The data support that threat from disease to the individual and its perception of vulnerability for disease might be used in prevention. Second, current smokers may have insufficient knowledge about the smoking-disease relationship. This is supported by the finding that only 3.4 % (127) of the ever smokers reported that they had received smoking cessation counseling from a physician during the last 12 months prior to the health examination. There could be a recall bias among the smokers, i. e. denying counseling which in fact has been provided. If it had not been provided this would indicate a shortcoming of health care. The majority had had contact to a physician in the last 12 months prior to the interview, and ever smokers who had consulted a physician during the last 12 months prior to the health examination were less likely to be current smokers than subjects who did not consult a physician during the last 12 months prior to the health examination. This result may indicate that having contact alone with a physician might add to stopping. However, those subjects who stated that they had received smoking cessation counseling had threefold odds to smoke at the time of the interview compared to those who mentioned that they did not have received cessation counseling. This might indicate that there is a subpopulation of hard core smokers who seem to have particular difficulty to stop. Nicotine dependence or single aspects of it, such as particularly strong craving for nicotine, might be factors that could be part of the resistance to smoking cessation. Evidence revealed that physician advice may increase the likelihood of stopping smoking [16]. However, it appears that much more may be done in the medical field to improve smoking cessation intervention. Third, a person may decide to smoke, fully aware of the smoking-disease relationship and hazarding the consequences.

A strength of the study is its representativeness for one nation although the country showed only very little activity in the prevention of tobacco-attributable death and disease at the time of the data collection. On the other hand, there are several limitations to the findings. First, this is a cross-sectional study, and the data do not allow any conclusions about causal relations. Second, our data do not reveal whether the threat from illness or vulnerability or a combination of these might be an origin of the smoking cessation. Third, the questionnaire did not include psychiatric comorbidity and nicotine dependence that may add to the maintenance of smoking in spite of the awareness of disease. Fourth, except for the BMI, data were based on self-statements only, and no validation according to smoking and according to circulatory disorders was used. However, evidence shows that the proportion of smokers who deny or minimize smoking in survey studies may be negligible because they do not significantly change the results with respect to smoking status [17]. According to circulatory disorders, only the awareness to have a specified circulatory disorder was of interest. We assumed this to be relevant for the intention to stop smoking.

## Conclusion

Substantial proportions of ever smokers report smoking in spite of their awareness of circulatory disorders. The data suggest that only a portion of smokers among the general population seems be discouraged from smoking by circulatory disorders or its accompanying cognitive or emotional processes. Barriers to quitting among these severely handicapped smokers should be explored.

## Competing interests

The author(s) declare that they have no competing interests.

## Authors' contributions

UJ carried out the data analysis and wrote major parts of the first draft of the paper. MH obtained the data, introduced the idea of the data analysis, analyzed parts of the data and contributed parts of the text to the paper. CM, HV and AS assisted with the writing of the manuscript and in the interpretation of the results.

## Pre-publication history

The pre-publication history for this paper can be accessed here:



## Figures and Tables

**Table 1:** Circulatory disorders, medical treatment and smoking cessation counseling among ever smokers

	n	Ever smokers		Chi^2^	P	w
	
		Former smokers row %	Current smokers row %			
Total	3778	39.3	60.7			
Women	1554	34.0	66.0			
Men	2224	42.9	57.1	30.3	< 0.001	.09
Age						
18 – 29	670	11.8	88.2			
30 – 39	982	29.7	70.3			
40 – 49	778	41.5	58.5			
50 – 59	666	50.0	50.0			
60 – 69	443	64.8	35.2			
70 – 79	239	70.7	29.3	537.0	< 0.001	.34
Number of circulatory disorders						
0	2718	33.4	66.6			
1	698	47.9	52.1			
2	198	62.1	37.9			
3 – 8	164	72.0	28.0	175.0	< 0.001	.21
Central circulatory disorders						
Hypertension						
No ^†^	3037	35.3	64.7			
Yes	739	55.6	44.4	100.7	< 0.001	.16
Myocardial infarction						
No ^†^	3674	38.4	61.6			
Yes	101	70.3	29.7	40.9	< 0.001	.10
Other coronary artery disease						
No ^†^	3536	37.5	62.5			
Yes	239	65.3	34.7	70.3	< 0.001	.14
Heart failure						
No ^†^	3629	38.4	61.6			
Yes	140	60.7	39.3	27.3	< 0.001	.09
Stroke						
No ^†^	3721	38.8	61.2			
Yes	51	68.6	31.4	18.2	< 0.001	.07
Other cerebrovascular disease						
No ^†^	3705	38.8	61.2			
Yes	68	63.2	36.8	16.2	< 0.001	.07
Peripheral circulatory disorders						
Peripheral vascular disease						
No ^†^	3549	38.1	61.9			
Yes	222	56.8	43.2	29.7	< 0.001	.09
Venous thrombosis						
No ^†^	3652	38.6	61.4			
Yes	121	59.5	40.5	20.7	< 0.001	.08
Consulted a physician during the last 12 months						
No	453	30.0	70.0			
Yes	3320	40.5	59.5	19.0	< 0.001	.07
Inpatient treatment during the last 12 months						
No	3283	38.1	61.9			
Yes	486	46.7	53.3	12.8	< 0.001	.06
Smoking cessation counseling						
No	3651	39.9	60.1			
Yes	127	21.3	78.7	19.4	< 0.001	.07
Exercise						
No	1821	37.0	63.0			
2 hours per week or less	1259	41.2	58.8			
more than 2 hours per week	676	41.4	58.6	7.4	< 0.05	.04
Body mass index						
less than 25.00	1536	26.6	73.4			
25.00 – 29.99	1507	47.4	52.6			
30.00 or higher	717	49.2	50.8	178.4	< 0.001	.21
School education						
9 years or less	1584	42.1	57.9			
10 – 12 years	1612	36.4	63.6			
13 or more years	559	39.0	61.0	11.1	< 0.01	.05

Chi^2 ^Likelihood chi^2 ^test result.

w Effect size measure Cohen's w [13].

^† ^No or do not know.

**Table 2:** Adjusted associations of the number of circulatory disorders with being a current smoker compared to former smoker

	Current smoker	
	
	Odds ratio	Confidence interval*
Number of central circulatory disorders		
0	ref	
1	0.88	0.72 – 1.09
2 – 6	0.60	0.43 – 0.83
Number of peripheral circulatory disorders		
0	ref	
1	0.99	0.73 – 1.33
2	0.87	0.42 – 1.79
Consulted a physician during the last 12 months		
No ^†^	ref	
Yes	0.61	0.47 – 0.78
Inpatient treatment during the last 12 months		
No ^†^	ref	
Yes	0.78	0.62 – 0.98
Smoking cessation counseling^†^		
No	ref	
Yes	3.16	1.97–5.09
Heavy smoker		
No ^‡^	ref	
Yes ^§^	0.69	0.59–0.81
Exercise		
No	ref	
2 hours per week or less	0.54	0.46–0.65
more than 2 hours per week	0.46	0.37–0.57
Body mass index		
less than 25.00	ref	
25.00 – 29.99	0.58	0.49–0.70
30.00 or higher	0.53	0.43–0.67
School education		
9 years or less	ref	
10 – 12 years	0.73	0.62–0.87
13 or more years	0.59	0.46–0.76
Sex		
male	ref	
female	1.10	0.93–1.30
Age		
18 – 29	ref	
30 – 39	0.31	0.23–0.42
40 – 49	0.20	0.15–0.26
50 – 59	0.14	0.10–0.19
60 – 69	0.07	0.05–0.10
70 – 79	0.05	0.03–0.07

N = 3,474 ever smokers.

Logistic regression analysis, all variables in the table were entered into the model.

* 95 %.

Central circulatory disorders: hypertension, myocardial infarction, other coronary artery disease, heart failure, stroke, other cerebrovascular disease.

Peripheral circulatory disorders: peripheral vascular disease, venous thrombosis.

ref Reference category.

^† ^No or do not know.

^‡ ^19 or less cigarettes per day or occasional smoker.

^§ ^20 or more cigarettes per day.

## References

[B1] De Backer G, Ambrosioni E, Borch-Johnsen K, Brotons C, Cifkova R, Dallongeville J, Ebrahim S, Faergeman O, Graham I, Mancia G, Cats VM, Orth-Gomer K, Perk J, Pyorala K, Rodicio JL, Sans S, Sansoy V, Sechtem U, Silber S, Thomsen T, Wood D (2003). European guidelines on cardiovascular disease prevention in clinical practice: third joint task force of European and other societies on cardiovascular disease prevention in clinical practice (constituted by representatives of eight societies and by invited experts). Eur J Cardiovasc Prev Rehabil.

[B2] Hall S, Bishop AJ, Marteau TM (2003). Increasing readiness to stop smoking in women undergoing cervical screening: evaluation of two leaflets. Nicotine Tob Res.

[B3] Schmitz JM, Spiga R, Rhoades HM, Fuentes F, Grabowski J (1999). Smoking cessation in women with cardiac risk: a comparative study of two theoretically based therapies. Nicotine Tob Res.

[B4] Kastarinen M, Tuomilehto J, Vartiainen E, Jousilahti P, Nissinen A, Puska P (2002). Smoking trends in hypertensive and normotensive Finns during 1982-1997. J Hum Hypertens.

[B5] Gulliford MC (2001). Low rates of detection and treatment of hypertension among current cigarette smokers. J Hum Hypertens.

[B6] Hall S, Weinman J, Marteau TM (2004). The motivating impact of informing women smokers of a link between smoking and cervical cancer: the role of coherence. Health Psychol.

[B7] John U, Meyer C, Rumpf HJ, Hapke U (2003). Relation among stage of change, demographic characteristics,  smoking history, and nicotine dependence in an adult German population. Preventive Medicine.

[B8] Bellach BM, Knopf H, Thefeld W (1998). Der Bundes-Gesundheitssurvey [The German Health Survey. 1997/98]. Gesundheitswesen.

[B9] Thefeld W, Stolzenberg H, Bellach BM (1999). Bundes-Gesundheitssurvey: Response, Zusammensetzung der Teilnehmer und Non-Responder-Analyse [The Federal Health Survey: response, composition of participants and non-responder analysis]. Gesundheitswesen.

[B10] Schroeder E, Potthoff P, Reis U, Klamert A (1998). Erhebungsarbeiten im Bundes-Gesundheitssurvey [Data evaluation in the German Health Survey]. Gesundheitswesen.

[B11] The World Medical Association (2004). The World Medical Association Declaration of Helsinki. Ethical principles for medical research involving human subjects.

[B12] Aronne LJ (2002). Classification of obesity and assessment of obesity-related health risks. Obes Res.

[B13] Cohen J (1988). Statistical power analysis.

[B14] John U, Meyer C, Rumpf HJ, Schumann A, Dilling H, Hapke U (2005). No considerable long-term weight gain after smoking cessation: evidence from a prospective study. European Journal of Cancer Prevention.

[B15] Ussher MH, West R, Taylor AH, McEwen A (2000). Exercise interventions for smoking cessation (Cochrane review). In: The Cochrane library, Issue 3.

[B16] Silagy C, Stead LF (2004). Physician advice for smoking cessation (Cochrane review). In: The Cochrane library, Issue 1.

[B17] Vartiainen E, Seppala T, Lillsunde P, Puska P (2002). Validation of self reported smoking by serum cotinine measurement in a community-based study. Journal of Epidemiology and Community Health.

